# The Human Milk Oligosaccharide 2'-Fucosyllactose Shows an Immune-Enhancing Effect in a Cyclophosphamide-Induced Mouse Model

**DOI:** 10.4014/jmb.2211.11049

**Published:** 2022-12-13

**Authors:** Seon Ha Jo, Kyeong Jin Kim, Soo-yeon Park, Hyun-Dong Paik, Ji Yeon Kim

**Affiliations:** 1 Department of Nano Bio Engineering, Seoul National University of Science and Technology, Seoul 01811, Republic of Korea; 2 Department of Food Science and Technology, Seoul National University of Science and Technology, Seoul 01811, Republic of Korea; 3 Department of Food Science and Biotechnology of Animal Resources, Konkuk University, Seoul 05029, Republic of Korea

**Keywords:** 2'-Fucosyllactose, cyclophosphamide, splenocytes, immune enhancement, concanavalin A

## Abstract

The 2'-fucosyllactose (2'-FL) is the richest components in a human milk oligosaccharide. Several studies have reported that 2'-FL has beneficial effects in infants. However, there are few studies on its immune-enhancing effects. This research aimed to examine the immune-enhancing effect of 2'-FL on immunosuppression by cyclophosphamide (CCP) in ICR mice. Mice were orally administered distilled water or 0.5 mg/kg B.W. 2'-FL for 14 days. An immunocompromised mouse model was induced using CCP 80 mg/kg B.W. at 12-14 days. Using the CCP had effects on reducing their body weight, organ weight, spleen index, natural killer (NK) cell activity, and cytokines concentration and expression. This study also used concanavalin A-mediated T-cell proliferation to verify the immune-enhancing effects in the sample. Body weight, spleen index, organ weight, and cytokine levels were measured to estimate the immune-enhancing effects. The body weight at 14 days tended to increase, and the spleen weight and index significantly increased in the 2'-FL group compared to the CCP group. The NK cell activity increased in the 2'-FL group compared to the CCP group, but there was no significant difference. The concentration of interleukin (IL)-2 tended to recover in the 2'-FL group compared to the CCP group. The 2'-FL group showed a significant increase of IL-10 and IFN-gamma concentration compared to the CCP group. In addition, there was a trend of increased IL-10 mRNA expression compared to the CCP group. These results revealed that 2'-FL improved CCP-induced immunosuppression, suggesting that 2'-FL may have the potential to enhance the immune system.

## Introduction

Breast milk is a source of bioactive compounds for the healthy development of infants with relatively weak immunity [1]. Previous studies have shown that breastfed infants have a lower risk of infection and inflammation than formula-fed infants, and breastfeeding also decreases the risk of gastrointestinal disease, food allergy, autoimmune disease, and metabolic syndrome [2]. Human milk (HM) is made up of highly variable compositions and is believed to be evolutionarily optimized to meet the needs of infants of various ages and stages of development [3].

Human milk oligosaccharides (HMOs) are nondigestible carbohydrates that are the third most abundant solid component of HM after lactose and lipids [4]. HMOs play an important role as components in HM that support immunity in neonates [5]. In addition, they are major bioactive components of HM and have received increased attention in recent years because of their ability to modulate microbiota compositions and prevent pathogen adhesion and infection [6]. HMOs comprise more than 200 different oligosaccharides with a wide variety of structures [7]. All known individual components of HMOs are subdivided into three major categories: fucosylated neutral HMOs, sialylated acidic HMOs, and nonfucosylated neutral HMOs. The fucosylated neutral HMO 2'-fucosyllactose (2'-FL) is the most abundant oligosaccharide, composing up to 20% of total HMOs [8, 9]. Previous clinical studies have documented that infant formulas containing HMOs such as 2'-FL support immune function and lower inflammatory cytokine levels [10, 11].

CCP is a member of the oxazaphosphorine family of mustard-alkylating agents, which is primarily utilized as an alkylating agent in chemotherapy [12]. In addition, CCP is known as a cytotoxic drug that can suppress both humoral and cellular immunity [13]. It causes an immune suppressive effect by interfering with DNA replication and damaging both tumor and normal tissue, so it can be fatal [14, 15].

The immune system is made up of an innate response and an acquired response. When pathogens invade a tissue, the innate immune system establishes a proper immune response, including inflammatory responses such as the generation of proinflammatory cytokines and chemokines [16]. Macrophages, NK cells, and dendritic cells are activated by antigens and lead to the secretion of cytokines in that system [18]. Stimulated macrophages can remove antigens directly by phagocytosis and release toxic or proinflammatory cytokines such as tumor necrosis factor-α (TNF-α), IL-1β, and IL-6 [18]. In contrast, adaptive immune systems consist of T and B cells. The biological function of Th1 and Th2 cells is mainly related to cytokine production [19]. Proinflammatory cytokines and mediators, including nitric oxide (NO), IFN-γ, IL-6, and IL-12, can be increased by activating T and B lymphocytes.

Most studies have investigated HMOs for their ability to increase immunity as complex HMO mixtures. Sharon M Donovan *et al*. reported that 2'-FL has a greater effect on inhibiting inflammation than 6'-SL, which is another individual component in HMOs [20]. Additionally, few studies have demonstrated the immune-enhancing effects of 2'-FL as an individual HMO, and its mechanism of action in inhibiting inflammation and immune responses is not clear. Therefore, this study aimed to identify the immune-enhancing potential of 2'-FL in immunosuppressed mice induced by CCP through inflammatory cytokines and immune pathway-producing cytokines.

## Materials and Methods

### Materials

Dulbecco’s phosphate buffered saline (DPBS), fetal bovine serum (FBS), and penicillin‒streptomycin mixture were obtained from Biowest (France). Roswell Park Memorial Institute (RPMI) 1640 medium was obtained from Gibco-Life Technologies (USA). CCP, concanavalin A (ConA), and trypan blue were obtained from Sigma-Aldrich (USA). 2'-FL was kindly provided by Advanced Protein Technologies Corp. (Korea).

### Animals and Designs

**Fig. 1:**
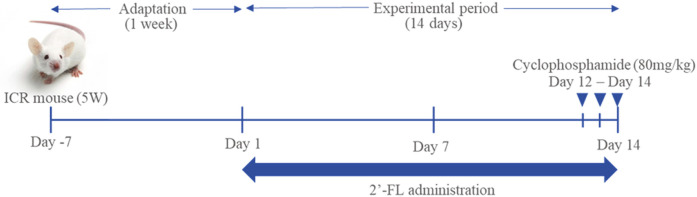
Experimental design. After an adaptation period of one week, 2'-FL was orally administered every day in the 2'-FL group for 14 days. CCP was administered to the CCP and 2'-FL groups intraperitoneally for 12 to 14 days before animal sacrifice.

Thirty male ICR mice (5 weeks old) were procured from Hana Biotech (Korea) and subjected to the experiment after a week of acclimation to the regular environment. The animal care and use protocol was reviewed and approved by the Institutional Animal Care and Use Committee (IACUC) at Southeast Medi-Chem Institute (Receipt Number: SEMI-21-006). After acclimatization, the animal models were randomly divided into three groups (*n* = 10). The divided groups were as follows: normal control (NC), CCP only (CCP), and CCP + 2'-FL (2'-FL). The NC and CCP groups were orally administered distilled water for 14 days. The mice in the 2'-FL groups were orally administered 2'-FL at concentrations of 0.5 g/kg B.W. for 14 days. The CCP group and 2'-FL group were intraperitoneally (i.p.) administered CCP diluted in saline (80 mg/kg B.W.) for 12 to 14 days to induce immunosuppression in animal models. The NC group mice were injected with saline during the same period. The animal experimental procedure is shown in Fig. 1.

### Natural Killer (NK) Cell Activity

At 15 days, whole blood was collected from all mice and mixed with NK cell-activating reagent. Then, the samples were incubated at 37°C for 24 h. After incubation, the collected supernatants were transferred to new tubes, and NK cell activity was measured by analyzing the IFN-γ production level using the Murine NK Activity Kit (NKMAX, Korea).

### Isolation of Splenocytes for Cell Culture

The spleen, mesenteric lymph nodes (MLNs), and Peyer's patches were washed with sterilized saline and weighed. The separated spleens were used to calculate the following index:

Spleen index (mg/g) = spleen weight (mg)/B.W. (g)

The spleens were filtered by using a syringe and cell strainer (SPL Life Sciences, Korea). Red blood cells were eliminated using 1 ml of red blood cell lysis buffer (Sigma-Aldrich, USA) and washed with RPMI 1640 medium. The isolated splenocytes were incubated in RPMI 1640 medium. They were seeded in 12-well plates at 1 × 10^6^ cells/well and incubated with 5 μg/ml ConA for 24 h at 37°C with 5% CO_2_. Supernatants and cell samples were harvested and stored in an aliquot at -80°C.

### Enzyme-Linked Immunosorbent Assay (ELISA)

For measurement of the levels of inflammatory cytokines in CCP-treated mouse splenocytes by ELISAs, the stored aliquoted supernatants were transferred to 96-well ELISA plates. The concentrations of the cytokines IL-2, IL-10, IL-12 receptor and IFN-γ were determined according to the R&D Systems (USA) DuoSet protocol.

### RNA Extraction and Quantitative Reverse Transcription Polymerase Chain Reaction (RT‒qPCR)

With TRIzol reagent (Life Technologies, USA), total RNA was extracted from splenocytes. The extracted RNA was reverse transcribed using a cDNA reverse transcription kit (Roche, Switzerland). RT-qPCR was performed using designed primers. Initial denaturation was performed for 30 s at 95°C, followed by 40 cycles at 95°C for 10 s and 60°C for 15 s. The Universal Probe Library (UPL) method was used to quantify the expression of IL-2, IL-10, and IL-12 receptor β1 in splenocytes using a LightCycler 96 system (Hoffmann La Roche). RT‒qPCR analysis was conducted using the sequences of the sense and antisense primers as follows: IL-2 (forward, 5´-gctgttgatggacctacagga-3´, reverse, 5´-ttcaattctgtggcctgctt-3´), IL-10 (forward, 5´-cagagccacatgctcctaga-3´; reverse, 5´-tgtccagctggtcctttgtt-3´), IL-12 receptor β1 (forward, 5´-ccccagcgctttagcttt-3´; reverse, 5´-gccaatgtatccgagactgc-3´), and glyceraldehyde 3-phosphate dehydrogenase (GAPDH) (forward, 5´-aagagggatgctgcccttac-3´; reverse, 5´-ccattttgtctacgggacga-3´). The relative mRNA expression, normalized to that of GAPDH, was calculated using the comparative CT method (delta delta CT).

### Statistical Analysis

The results are presented as the mean ± standard error of the mean (SEM). Statistical significance was analyzed using one-way ANOVA followed by Duncan’s multiple range test, and comparisons between groups were analyzed using Student’s t test (SAS 9.4, SAS Institute, USA). Data with *p* < 0.05 were considered statistically significant.

## Results

### Effects of 2'-FL on Body Weight and the Spleen Index of CCP-Treated Mice

To confirm the immune-enhancing effect of 2'-FL against immunosuppression by CCP, we measured the body weight on days 0, 7, and 14 (Table 1). On day 7, body weight increased in all groups compared with their body weight on day 0. On day 14 after the injection of CCP, the body weight of the NC group increased by 32.3% from 26.36 g to 34.88 g. However, the weights of the CCP and 2'-FL groups decreased to 30.19 g and 31.52 g on day 14, respectively.

**Fig. 2:**
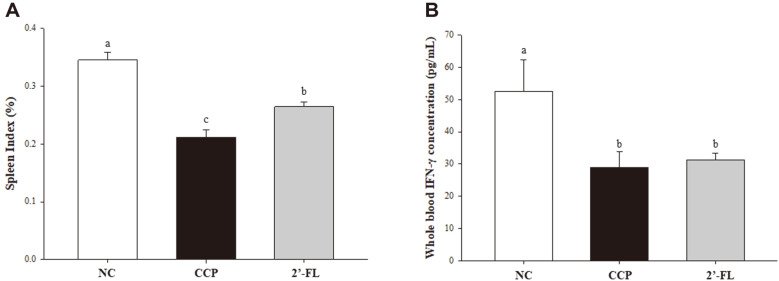
Effect of 2'-FL on the spleen index (A) and NK cell activity (B) in immunosuppressed mice. NC, Normal control; CCP, group induced by CCP (80 mg/kg B.W.); 2'-FL, group that received 2'-FL induced by CCP (80 mg/kg B.W. The data represent the mean ± SEM. Significant differences (*p* < 0.05) between the groups are indicated with different letters determined by Duncan’s multiple range test.

In terms of changes in spleen, MLN, and Peyer’s patch weight, the NC group showed the highest levels on these organ weights (Table 2). The spleen weight was significantly decreased in the CCP group compared with the NC group (*p* < 0.001). The 2'-FL group showed significant increases in spleen weight compared to the CCP group. Likewise, the spleen index was significantly decreased in the CCP group compared to the NC group (Fig. 2A). The spleen index of the 2'-FL group was significantly increased compared to that of the CCP group. The MLN weight of CCP group decreased compared to that of the NC group, and the weight of the 2'-FL group tended to increase but there was no significant difference compared to that of the CCP group (*p* = 0.071). The Peyer’s patch weight of the NC group was the highest, and there was no significant difference between the CCP and 2'-FL groups.

### Effects of 2'-FL on NK Cell Activity in the CCP-Induced Mouse Model

As shown in Fig. 2B, the IFN-γ concentration in whole blood NK cells was significantly decreased in the CCP group (28.40 ± 5.40 pg/ml) compared to the NC group (53.19 ± 10.20 pg/ml). The NK cell activity level tended to increase by treatment with 2'-FL (30.41 ± 2.23 pg/ml), but there was no significant difference.

### Effects of 2'-FL on the Levels of Inflammatory Cytokines in CCP-Treated Mouse Splenocytes

**Fig. 3:**
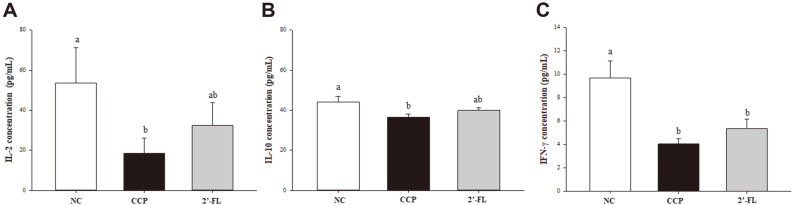
Effect of 2'-FL on the production of the inflammatory cytokines IL-2 (A) IL-10 (B), and (C) IFN-γ by immunosuppressed mice. NC, Normal control; CCP, group induced by CCP (80 mg/kg B.W.); 2'-FL, group that received 2'- FL induced by CCP (80 mg/kg B.W. The data represent the mean ± SEM. Significant differences (*p* < 0.05) between the groups are indicated with different letters determined by Duncan’s multiple range test.

The levels of inflammatory cytokines in splenocytes treated with ConA are presented in Fig. 3. CCP injection affected all the analyzed cytokine levels, causing a significant reduction in IL-2, IL-10, and IFN-γ compared to those of the NC group. The concentration of IL-2 tended to increase approximately 1.74-fold in the 2'-FL group compared with the CCP group (*p* = 0.073). The IL-10 concentration tended to increase approximately 1.08-fold in the 2'-FL group compared with the CCP group. In addition, the IFN-γ concentration of the 2'-FL group was increased by approximately 1.32-fold compared with that of the CCP group, but there was no significant difference.

### Effects of 2'-FL on the mRNA Expression of IL-2, IL-10, and IL-12 Receptor β1 (IL-12r) in CCP-Induced Mouse Splenocytes

**Fig. 4:**
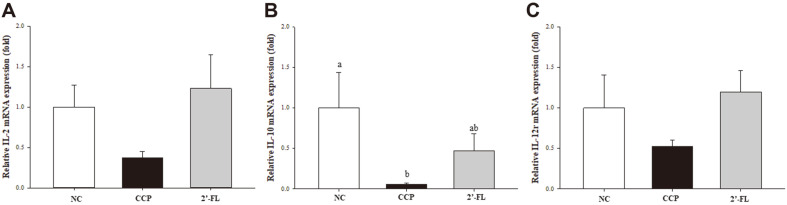
Effect of 2'-FL on the expression levels of the inflammatory cytokines IL-2 (A), IL-10 (B), and (C) IL- 12r in immunosuppressed mice. NC, Normal control; CCP, group induced by CCP (80 mg/kg B.W.); 2'-FL, group that received 2'-FL induced by CCP (80 mg/kg B.W. The data represent the mean ± SEM. Significant differences (*p* < 0.05) between the groups are indicated with different letters determined by Duncan’s multiple range test. An absence of a letter in all groups indicates that there is no significant difference.

The relative mRNA expression of IL-2, IL-10, and IL-12r in ConA-stimulated splenocytes isolated from the CCP-treated mice was measured by RT‒qPCR (Fig. 4). The expression in the CCP group decreased by approximately 36.9% compared to that in the NC group (*p* = 0.051), and the 2'-FL group showed an increase of approximately 3.32-fold in relative mRNA expression of IL-2 compared to the CCP group, but there was no significant difference. In terms of IL-10 mRNA expression, the CCP group showed a 94.4% reduction compared with the NC group (*p* = 0.064), and the 2'-FL group showed recovery compared with the CCP group, but there was no significant difference. The relative IL-12r mRNA expression was decreased by approximately 52.7% in the CCP group compared to the NC group, although there was no significant difference.

## Discussion

HM has been regarded as the only source of HMOs, but 2'-FL, an individual type of HMO, has been recently synthesized and shown to be structurally and functionally identical to that in HM [10]. Thus, 2'-FL in HMOs is now available in some commercial infant formulas in addition to nondigestible carbohydrates (NDCs), such as galactooligosaccharide and fructooligosaccharide. Some studies have shown variable health benefits of these formulas, including immunomodulatory effects and reduction in the incidence of infections [11, 21]. To our knowledge, this is the first study to use immunosuppressed animal models induced by CCP to confirm the immune enhancement of 2'-FL.

CCP, an alkylating cytotoxic agent with an immunosuppressive effect, was intraperitoneally administered to induce immunosuppression to confirm the immune-enhancing effects of 2'-FL. The CCP-induced immunosuppressive mouse model could mimic the immunocompromised state. According to a previous study, CCP can lead to a decrease in body weight, organ index, NK cell activity, and immune cell proliferation [22], which was confirmed in this study via a noticeable decrease in body weight, organ weight (spleen, MLN, Peyer’s patch), and spleen index. CCP interferes with the proliferation and differentiation of lymphocytes of T and B cells, suppressing the immune system [23]. The effects of immune repression on mice were also reflected by the spleen index because of the important roles of these organs in immunity. The spleen is one of the major organs that contains many immune cells, such as monocytes, lymphocytes, macrophages, and NK cells [24]. The spleen index was significantly decreased by approximately 50% in the CCP group compared to the NC group in this study. Also, a previous study reported that the destruction of splenocytes and thymocytes was also observed in the histopathological sections of mice treated with CCP [25]. However, the 2'-FL group in this study showed inhibited immunosuppressive effects of CCP on immune-related organ weight and spleen index. The weights of the spleen and mesenteric lymph nodes in the 2'-FL group were increased by 1.35- and 1.15-fold, respectively, compared to those in the CCP group. However, the weight of Peyer’s patches in the 2'-FL group was lower than that in the other groups, and it is possible that the immunosuppressive effect of CCP had a greater effect on this group than the effect of 2'-FL on immune enhancement. The spleen index in the 2'-FL group increased by 1.25-fold compared to that in the CCP group. This result indicates that 2'-FL leads to improved cellular and humoral immune responses, which suggests that 2'-FL shows potential for restoration of the immune system.

NK cells are large granular lymphocytes related to innate immune mechanisms. They are known to recognize and immediately eliminate abnormal cells such as cancer cells or virus-infected cells [26]. These cells are abundant in the blood, spleen, and liver under homeostatic conditions [27] and play an important role in the first phase of host defense against infections. During infection, myeloid cells contribute to activating several immune cells, such as NK cells [28]. They also mediate the acquired immune response through interaction with other immune cells. Recent studies have provided evidence for the role of NK cells in inflammation and the innate response by secreting inflammatory cytokines such as IFN-γ and TNF-α [29, 30]. IFN-γ produced by NK cells can reflect NK cell activity levels, so it can be an indicator of immune systems. In addition, whole blood we used for measuring the IFN-γ level could show the actual immune status in the body [31]. Thus, in this study, the IFN-γ concentration in whole blood was investigated to demonstrate immunosuppression by CCP and the immune-enhancement effect of 2'-FL. There was an increase of 1.08-fold in the 2'-FL group compared to the CCP group although there was no significant difference in NK cell activity. Because a previous study reported that CCP has an antitumor immunity effect via immune potentiation or immediate cytolytic activity, immune potentiation could be induced by injection of CCP [32].

ConA enhances the immune response of splenocytes in CCP-induced mouse models. ConA is a mitogen that is derived from lectins of plant proteins [33]. It is a small bioactive protein that nonspecifically activates mature T-cell proliferation and lymphokine production in a TCR-dependent manner by aggregating multiple cell surface glycoproteins [34]. When helper T (Th) cells contact antigen-presenting cells such as macrophages, they protect the body from infection, and they secrete both proinflammatory cytokines and anti-inflammatory cytokines [35]. In this research, we were able to confirm the cytokine indicator levels by using ConA to induce T-cell differentiation and a corresponding immune response. CD4+ Th cells can differentiate into Th1 cells or Th2 cells depending on the cytokine environment. IL-2, IFN-γ, and other inflammatory cytokines promote the differentiation of Th cells to the Th1 phenotype [36]. In splenocytes, 2'-FL tended to upregulate the mRNA expression of the cytokines IL-2, IL-10, and IL-12r from T cells by 3.32-, 8.08-, and 2.27-fold, respectively, compared to those of the CCP group. Similarly, Ignasi Azagra-Boronat *et al*. reported that intake of 2'-FL enhanced the immune system through the regulation of inflammatory cytokine levels, such as IL-4, IL-6, IL-10, IL-12p70, TNF-α, and IFN-γ, in the gut [37, 38]. In addition, the 2'-FL group showed an increase in the Th1/Th2 ratio, indicating increased activation of the Th1 response [37, 38]. IL-10 is an anti-inflammatory cytokine that inhibits the proinflammatory reactions of CD4+ Th cells. The 2'-FL groups showed increased levels of IL-10, and other studies also showed that ingesting 2'-FL can lead to stimulation of immune responses and inhibition of inflammation [39]. Anti-inflammatory cytokines such as IL-4 differentiate Th cells into an immunosuppressive Th2 phenotype, and IL-10 inhibits Th1 cytokine synthesis [40]. Th2 cells act on B cells and can induce antibody production as well as differentiation into memory B cells [41]. They can recruit eosinophils and mast cells by producing cytokines such as IL-4, IL-6, IL-10 and IL-13. IL-12r mRNA expression was significantly increased in the 2'-FL group compared with the CCP group. This method would have the potential to enhance NK cell activity accompanied by upregulation of IFN-γ and IL-2. In the present study, 2'-FL showed a tendency to increase the cytokine concentrations from T cells (IL-2, IL-10, and IFN-γ) compared to those of the CCP group by 1.74-, 1.08-, and 1.32-fold, respectively. Therefore, 2'-FL can promote innate immune systems through the stimulation of T cells, which would enhance inflammatory cytokine expression on lymphocytes.

Several results in the study demonstrated that 2'-FL inhibits infection and inflammation, showing a reduction in inflammatory markers in vitro and in vivo [42, 43]. Conversely, this study used animal models induced by CCP and showed immune enhancement of 2'-FL to be more effective than in other studies, which usually showed a reduction in inflammatory markers after administration of HMOs [42-44]. However, in this study, the detailed mechanisms of the innate immune response involving inflammatory cytokines and signal transduction pathways were not clearly clarified. Since we mostly explained the effect of 2'-FL through T-cell activation, immune studies through B-cell activation should be conducted along with T-cell activation. Also, further studies using other mitogens, such as LPS, are required to investigate the precise mechanisms and gain more data about other HMOs and the whole complex HMO mixture [45]. In conclusion, 2'-FL can induce T-cell activation and consequent innate immunity enhancement, which can be helpful in future research, such as formula preparation for immune enhancement.

## Figures and Tables

**Table 1:** Effect of 2'-FL on the body weight of immunosuppressed mice.

Group	Body weight (g)		

	Day 0	Day 7	Day 14
NC	26.36 ± 0.18	33.91 ± 0.19	34.88 ± 0.32^a^
CCP	26.28 ± 0.19	32.57 ± 0.30	30.19 ± 0.45^b^
2'-FL	26.32 ± 0.15	33.48 ± 0.43	31.52 ± 0.45^b^

NC, Normal control; CCP, group induced by CCP (80 mg/kg B.W.); 2'-FL, group that received 2'-FL induced by CCP (80 mg/kg B.W. The data represent the mean ± SEM. Significant differences (*p* < 0.05) between the groups are indicated with different letters determined by Duncan’s multiple range test. An absence of a letter in all groups indicates that there is no significant difference.

**Table 2:** Effect of 2'-FL on the Spleen, Mesenteric lymph node, and Peyer’s patch weight of immunosuppressed mice.

Group	Spleen (g)	Mesenteric Lymph Node (g)	Peyer’s Patch (g)
NC	0.1261 ± 0.0132^a^	0.2286 ± 0.0214^a^	0.0685 ± 0.0067^a^
CCP	0.0629 ± 0.0115^c^	0.1898 ± 0.0122^a^	0.0357 ± 0.0087^b^
2'-FL	0.0847 ± 0.0081^b^	0.2179 ± 0.0075^ab^	0.0286 ± 0.0046^b^

NC, Normal control; CCP, group induced by CCP (80 mg/kg B.W.); 2’-FL, group received 2'-FL induced by CCP (80 mg/kg B.W.); The data represents mean ± SEM. Significant differences (*p* < 0.05) between the groups are indicated with different letters determined by Duncan’s multiple range test.
